# Seasonal Serum 25(OH) Vitamin D Level and Reproductive or Immune Markers in Reproductive-Aged Women with Infertility: A Cross-Sectional Observational Study in East Japan

**DOI:** 10.3390/nu15245059

**Published:** 2023-12-09

**Authors:** Kuniaki Ota, Junichiro Mitsui, Shoko Katsumata, Yuko Takayanagi, Yurie Nako, Makiko Tajima, Akira Komiya, Toshifumi Takahashi, Kiyotaka Kawai

**Affiliations:** 1 Fukushima Medical Center for Children and Women, Fukushima Medical University, 1 Hikarigaoka, Fukushima 960-1295, Japan; totakaha@fmu.ac.jp; 2 Department of Obstetrics and Gynecology, Tokyo Rosai Hospital, Japan Labor Health and Safety Organization, 4-13-21 Ohmori-minami, Ohta-ku, Tokyo 143-0013, Japan; 3 Reproductive Medicine, Kameda IVF Clinic Makuhari, Makuhari Techno Garden D3F, 1-3 Nakase, Mihama-ku, Chiba-City 261-8501, Japan; jmticrm@tmd.ac.jp (J.M.); katsumata.shoko@kameda.jp (S.K.); takayanagi.yuko@kameda.jp (Y.T.); nako.yurie@kameda.jp (Y.N.); tajima.makiko@kameda.jp (M.T.); komiya.akira@kameda.jp (A.K.); kawai.kiyotaka@kameda.jp (K.K.); 4 Comprehensive Reproductive Medicine, Graduate School of Medical and Dental Sciences, Tokyo Medical and Dental University, 1-5-45 Yushima, Bunkyo-ku, Tokyo 113-8519, Japan

**Keywords:** vitamin D, infertility, reproduction, conception, ovarian reserve, fertility, immune markers, follicle-stimulating hormone, anti-Müllerian hormone, Th1/Th2

## Abstract

Several studies have reported that vitamin D may modify human reproductive functions; however, the results are conflicting. We aimed to comprehensively evaluate serum vitamin D levels and examine the relationship between serum vitamin D levels and ovarian reserve markers, and immune markers of implantation, in reproductive-aged Japanese women with infertility.in reproductive-aged women with infertility. This cross-sectional, single-center study included reproductive-aged women who underwent preconception screening for fertility. Serum vitamin D levels and reproductive and immune markers were measured. Standard and advanced statistical techniques were used. We observed a statistically significant difference in the seasonal and monthly 25(OH) vitamin D levels; the 25(OH) vitamin D level during winter was the lowest among all seasons. However, there was no linear correlation between 25(OH) vitamin D levels and ovarian reserve markers, such as follicle-stimulating hormone and anti-Müllerian hormone, or the Th1/Th2 cell ratio, which is used as an implantation-related immunological marker. In this large-scale study, we evaluated the serum 25(OH) vitamin D concentration in reproductive-aged women with infertility in Japan; however, there was no association between reproductive function and vitamin D levels.

## 1. Introduction

Vitamin D is essential for regulating bone and calcium metabolism, as well as maintaining muscle strength and immune function [1,2]. Vitamin D also positively affects female fertility by improving ovarian and endometrial physiology [3]. Serum vitamin D concentration is positively associated with ovarian reserve markers, such as follicle-stimulating hormone (FSH) [4] and anti-Müllerian hormone (AMH) [5]. Endometrial receptivity for embryonic implantation may be lower in women with hypovitaminosis D [6]. Moreover, low pre-conception vitamin D levels are associated with poor clinical outcomes in women undergoing in vitro fertilization (IVF) [7,8], recurrent pregnancy loss (RPL) [7,9,10], and recurrent implantation failure (RIF) [11].

Vitamin D plays a pivotal role in the regulation of immune responses by promoting T-helper (Th) and suppressing Th1 responses [12,13,14,15,16,17]. Key players in the maternal immune response to the embryo and fetus are cytokines produced by Th cells. Successful pregnancy requires a balance between the pro- and anti-inflammatory cytokines secreted by Th1 and Th2 cells [18,19,20]. Indeed, the levels of Th1 and Th2 cells were significantly higher and lower, respectively, in women with a history of RIF and RPL compared to those in fertile women, resulting in a significantly higher Th1/Th2 cell ratio [21,22]. Taken together, the vitamin D status may be strongly associated with conception rates in reproductive-aged women [9,10,23].

Vitamin D status is been focused on since vitamin D insufficiency is a common problem all over the world [24]. Most clinicians are concerned about the clinical significance of vitamin D because of the high rates of vitamin D deficiency and insufficiency. One considerable issue is the lack of consensus on the definition of Vitamin D sufficiency because Vitamin D insufficiency is defined as serum 25(OH) vitamin D (25(OH)D) concentration < 75 nmol/L (<30 ng/mL) and vitamin D deficiency as serum 25(OH)D concentration < 50 nmol/L (<20 ng/mL); 25(OH)D is considered to be a reliable indicator of vitamin D status in the body [1,25]. According to this definition, which is applied in most countries, there is a high prevalence (50–95%) of vitamin D insufficiency in the normal North American population [26,27], and similar observations have been described in East Asians [28,29], including the Japanese population [30,31]. Although vitamin D has been reported to play a key factor in oocyte development, embryo quality, endometrial receptivity, and human reproduction at physiological levels, it is currently debatable whether vitamin D is a valuable marker for women with infertility [32]. Despite this controversy, few large-scale studies have examined the prevalence of low blood vitamin D levels in women with infertility and the direct association of vitamin D with reproductive function.

We aimed to comprehensively investigate the evaluation of serum vitamin D levels in Japanese women with infertility and analyze the association between serum vitamin D levels and ovarian reserve markers, and immune markers of implantation, in reproductive-aged women with infertility.

## 2. Materials and Methods

### 2.1. Study Site

This retrospective study was undertaken at the Kameda IVF Clinic Makuhari (Chiba, Japan), located in Japan at a latitude of 35.607° N. This location received an average of 1945.5 h of daylight per year during the research period (https://www.data.jma.go.jp accessed on 1 April 2023).

### 2.2. Study Design and Participants

We reviewed 2029 women with infertility, which is defined as 1 year of unwanted non-conception with unprotected intercourse in the fertile phase of the menstrual cycle as a couple [33], who provided blood samples at their pre-conception checkup with the first blood test, including 25(OH)D, and completed a behavioral interview between September 2016 and December 2021. Information regarding the factors that potentially influence vitamin D status was obtained as follows: age, body mass index (BMI), gravity, parity, duration of infertility, causal factors of infertility, basal serum FSH, luteinizing hormone (LH), estrogen concentration on day 3 of the menstrual cycle, serum AMH, serum thyroid-stimulating hormone (TSH), serum free thyroxine (FT4), the use of vitamin D supplements, smoking status, and occupation status were retrieved from medical records. The first blood test sample was used to analyze the association between vitamin D status and these factors. According to definitions set by the Japan Metrological Agency, the season at the time of measurement was categorized for 3 months and recorded as “spring” (March–April–May), “summer” (June–July–August), “autumn” (September–October–November), and “winter” (December–January–February); the participants were divided into four groups using this categorization. In addition, AMH and FSH levels were analyzed as continuous and categorical variables divided into four quartiles at the first quartile (<25th percentile), second quartile (25th to ≤50th percentile), third quartile (50th to ≤75th percentile), and fourth quartile (>75th percentile).

#### 2.2.1. Measurement of 25(OH) Vitamin D

Serum 25(OH)D levels were measured via liquid chromatography–tandem mass spectrometry. The measurements were performed at LSI Medience Corporation (Tokyo, Japan). To calibrate and validate the measurements, the company used the 6PLUS1 25 OH-Vitamin D3 and D2 serum control, two-stage (I + II), according to the company’s application notes. These materials were verified to National Institute of Standards and Technology standards and were supplied by Chromsystems (Gräfelfing, Germany). The lower limit of quantitation was set at <1.0 ng/mL for 25(OH)D. The intra-assay variation was ≤6% for 25(OH)D over the concentration range, and the inter-assay variation was ≤11%. Serum 25(OH)D levels were classified as deficient, insufficient, or sufficient based on values < 20.0 ng/mL, 20–30 ng/mL, and ≥30.0 ng/mL, respectively [25,34].

#### 2.2.2. Th1/Th2 Ratio Analysis

Immunoassays were performed on IFN-γ producing Th cells (Th1 cells; CD4+ T lymphocytes with IFN-γ without IL-4) and IL-4 producing Th cells (Th2 cells; CD4+ T lymphocytes with IL-4 without IFN-γ) at SRL Inc, Japan, as previously mentioned [35]. For Th cell levels, blood samples were analyzed via laser flow cytometry (Fascinator II; BD Biosciences) on the day of collection with the use of brefeldin-A, ionomycin, phorbol 12-myristate 13-acetateycin (Sigma-Aldrich Corp. St Louis, MO, USA), FACS Permeabilizing Solution 2 (BD Biosciences, Tokyo, Japan), CD4 phycoerythrin-cyanine (PC)-5 (Immunotec, Marseille, France), FastImmune IFN-, fluorescein isothiocyanate (FITC)/IL-4 PE (BD Biosciences), and Fluorescence Activated Cell Sorting (FACS) Lysing Solution (BD Biosciences). Erythrocytes were lysed, and specific intracellular staining was performed using FastImmune IFN-FITC/IL-4-PE (Becton Dickinson Biosciences, San Jose, CA, USA), according to the manufacturer’s instructions, after the surface staining of activated whole blood samples with an anti-CD4-PC5 monoclonal antibody. The ratio of IFN-γ-positive to IL-4-positive Th cell levels was utilized to calculate the Th1/Th2 cell ratio and indicate a more pro- or anti-inflammatory composition of the T cell compartment [36].

### 2.3. Ethical Statement

This study received the approval of the ethics review committee of Kameda IVF Clinic Makuhari (22-042). After a detailed description of the purpose of this study, written informed consent was obtained from all participants. All experimental procedures were conducted according to the tenets of the Declaration of Helsinki.

### 2.4. Statistical Analyses

Continuous variables are reported and analyzed using descriptive tests such as means, SDs (standard deviations), or medians (interquartile range (IQR)). Kolmogorov–Smirnov tests were performed to assess the normality of the variables before further statistical analyses. The Mann–Whitney U test or Kruskal–Wallis test and/or Jonckheere–Terpstra test was performed. Categorical data are presented as counts and percentages and compared using a one-way analysis of variance or McNemar’s chi-square test. Pearson’s correlation was used to illustrate the linearity between 25(OH)D levels and AMH, FSH, and the Th1/Th2 cell ratio. The data were analyzed using EZR version 1.51 statistical software [37], and significance was set at *p* < 0.05.

## 3. Results

### 3.1. Population Characteristics

The clinical characteristics of the 2029 infertile women included in this study are presented in Table 1. The mean age of the participants was 34.5 ± 4.5 (range: 22–47) years. Gravity and parity were 0.5 ± 0.9 (range: 0–8) and 0.2 ± 0.5 (range: 0–4), respectively. The BMI ranged from 14.8 to 40.9 kg/m^2^ (mean, 21.6 ± 3.5 kg/m^2^). The duration of infertility was 22.7 ± 18.5 (range: 0–120) months. The percentage of causal factors of infertility were reduced ovarian reserve, 20.7% (*n* = 421), ovulation disorder, 24.1% (*n* = 488), uterine factor, 40.8% (*n* = 827), tubal factor, 18.2% (*n* = 369), endometriosis, 11.7% (*n* = 238), male factor, 42.2% (*n* = 857), and unexplained, 14.6% (*n* = 297). Baseline serum FSH, LH, and estrogen on day 3 of the menstrual cycle were 8.2 ± 5.2 (range: 0.1–85.9) IU/mL, 7.4 ± 5.7 (range: 0.1–61.4) IU/mL, and 50.8 ± 59.8 (range: 5.0–354) pg/mL, respectively. Mean AMH was 3.6 ± 3.1 ng/mL (range:0.2–48.2). Thyroid-stimulating hormone and free thyroxine were 1.7 ± 1.1 (range: 0.1–13.5) μIU/mL and 1.3 ± 0.2 (range: 0.5–3.8) ng/dL, respectively. Among the participants, 10.8% used Vitamin D supplements, and 15.2% were current smokers.

### 3.2. Serum 25(OH)D Levels in Reproductive-Aged Women with Infertility

**Figure 1:**
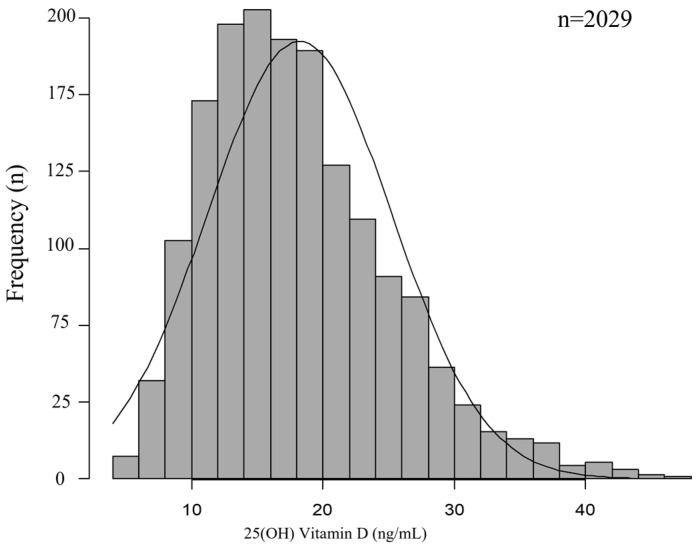
Frequency distribution of 25(OH) vitamin D.

**Figure 2:**
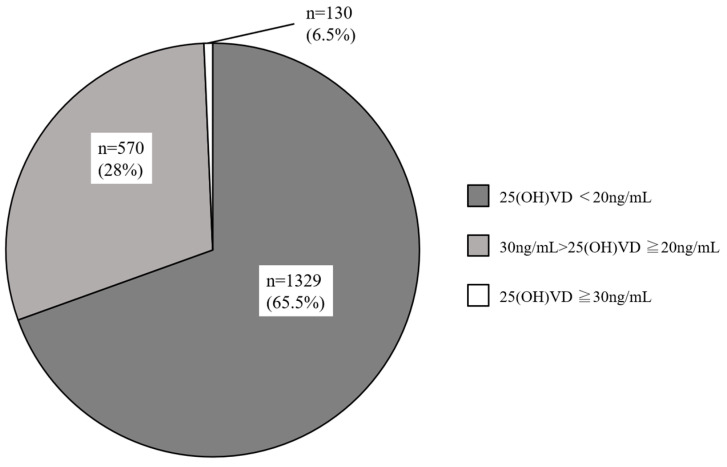
Percentage of infertile women with 25(OH) vitamin D status.

Figure 1 illustrates the frequency distribution of serum 25(OH)D concentrations in the 2029 women with infertility, and serum 25(OH)D concentrations were normally distributed (D = 0.067696, *p* < 0.01). They were divided into three categories based on Endocrine Society, Japan Society for Endocrinology, and the Institute of Medicine guidelines, with vitamin D levels categorized as sufficient (25(OH)D  ≥  30 ng/mL), insufficient (25(OH)D  =  20–29 ng/mL), or deficient (25(OH)D  <  20 ng/mL) [34,38,39]. The mean serum 25(OH)D level was 18.2 ± 7.0 ng/mL (in the range of 4.1–46.6 ng/mL) mL, and 65.5% of the participants were deficient (<20 ng/mL), 28.0% were insufficient (20–29 ng/mL), and only 6.5% of the subjects were sufficient (≥30 ng/mL) (Figure 2).

#### 3.2.1. Serum 25(OH)D Levels and Reproductive Parameters

The correlation between the 25(OH)D concentration and clinical characteristics was statistically analyzed (Table 2). No significant correlation was observed between 25(OH)D concentration and age, BMI, AMH, FSH, or TSH levels. The season of blood collection significantly correlated with 25(OH)D concentration (Table 2).

#### 3.2.2. Seasonal and Monthly Serum 25(OH)D Levels

**Figure 3:**
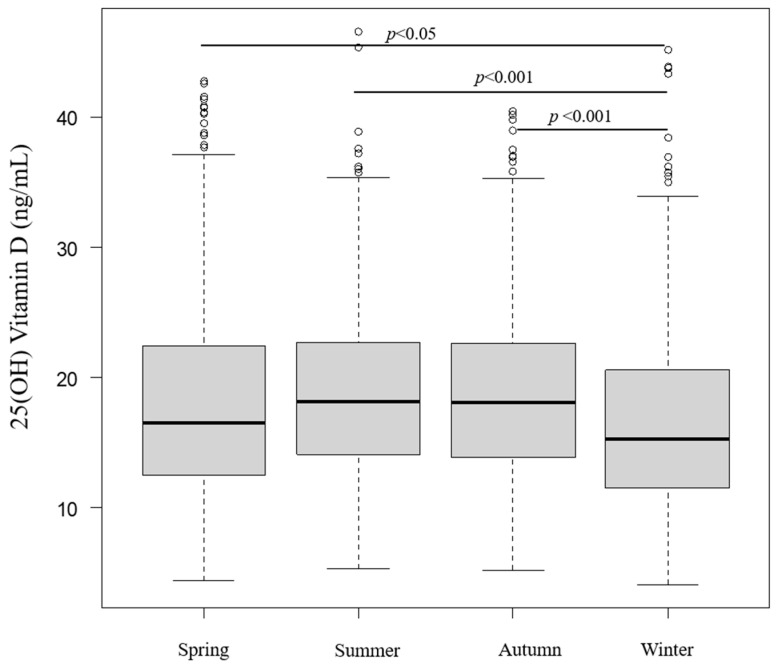
Seasonal status of 25(OH) vitamin D.

**Figure 4:**
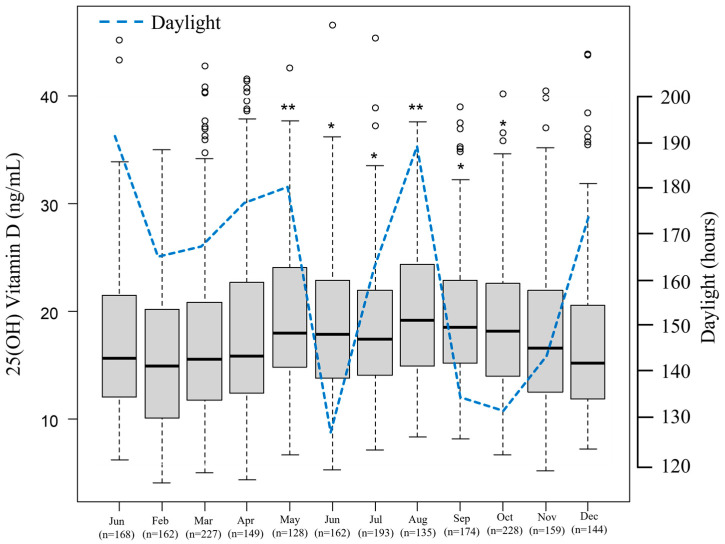
25(OH)Vitamin D concentrations based on month of serum collection. * *p* < 0.05, and ** *p* < 0.001 compared with February (one-way ANOVA test).

Seasonal 25(OH)D levels were statistically analyzed and revealed significantly higher 25(OH)D levels in seasons other than winter (spring vs. winter: *p* < 0.05, summer vs. winter: *p* < 0.001, autumn vs. winter: *p* < 0.001) (Figure 3). Figure 4 depicts the monthly serum 25(OH)D concentrations. The highest 25(OH)D concentration was observed in August and the lowest in February. Serum 25(OH)D concentrations in May, June, July, August, September, and October were significantly higher than those in February.

#### 3.2.3. Seasonal Status of Serum 25(OH)D Levels and Reproductive Markers Categorized as Ovarian Reserve

**Figure 5:**
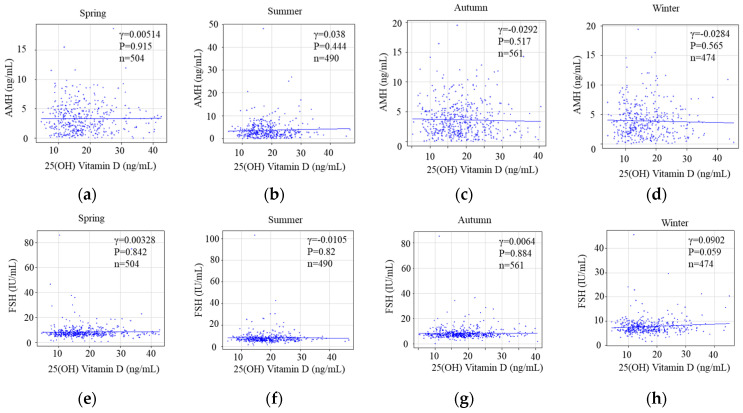
The correlation of seasonal 25(OH)Vitamin D versus reproductive markers of ovarian reserves with AMH and FSH. Upper: The correlation between AMH and 25(OH) vitamin D in four seasons; spring (**a**), summer (**b**), autumn (**c**), and winter (**d**) visualized by a scatter plot in a linear scale. Lower: The correlation between FSH and 25(OH) vitamin D in four seasons; spring (**e**), summer (**f**), autumn (**g**), and winter (**h**) visualized by a scatter plot in a linear scale.

A statistical analysis of seasonal 25(OH)D levels and ovarian reserves revealed no significant correlation between seasonal 25(OH)D concentrations and AMH (spring: Pearson r = 0.00514, *p* = 0.915; summer: Pearson r = 0.038, *p* = 0.444; autumn: Pearson r = −0.0292, *p* = 0.517; winter: Pearson r = −0.0284, *p* = 0.565) (Figure 5a–d). Moreover, FSH levels did not correlate with 25(OH) D concentrations during any season (spring: Pearson r = 0.00328, *p* = 0.842; summer: Pearson r = −0.0105, *p* = 0.82; autumn: Pearson r = 0.0064, *p* = 0.884; winter: Pearson r = 0.0902, *p* = 0.059) (Figure 5e–h).

#### 3.2.4. Serum 25(OH)D Levels or the Degree of Deviation of Serum 25(OH)D Levels and the Status of Helper T-Cell Immunity as an Implantation Marker

**Figure 6:**
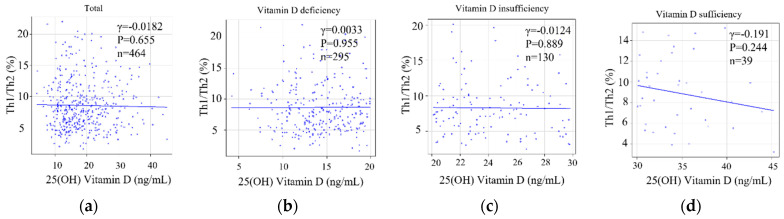
The correlation of degree of deviation of 25(OH)vitamin D level versus implantation marker with Th1/Th2 cell ratio. The correlation between AMH and populations of 25(OH) vitamin D; total (**a**), vitamin D deficiency (**b**), vitamin D insufficiency (**c**), and vitamin D sufficiency (**d**) visualized by a scatter plot in a linear scale.

Serum 25(OH)2D levels and the Th1/Th2 cell ratio were not correlated in the 464 reproductive-aged women with infertility (Pearson r = −0.0182, *p* = 0.695) (Figure 6a). There were no significant differences in the serum 25(OH)D level or Th1/Th2 cell ratio among deficient (Pearson r = 0.0033, *p* = 0.955) (Figure 6b), insufficient (Pearson r = −0.0124, *p* = 0.889) (Figure 6c), and sufficient (Pearson r = −0.192, *p* = 0.244) (Figure 6d) groups.

**Figure 7:**
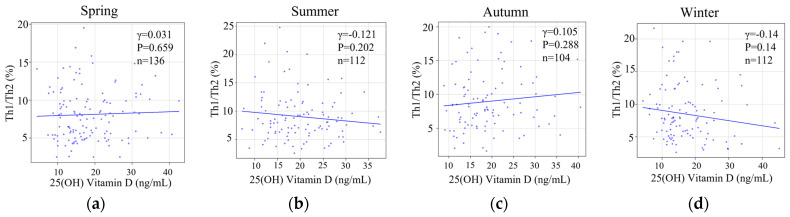
The correlation of seasonal 25(OH)Vitamin D versus implantation marker with Th1/Th2 cell ratio. The correlation between 25(OH) vitamin D and Th1/Th2 cell ratio in four seasons; spring (**a**), summer (**b**), autumn (**c**), and winter (**d**) visualized by a scatter plot in a linear scale.

Furthermore, the Th1/Th2 cell ratio was not correlated with serum 25(OH)D levels in spring (Pearson r = −0.0381, *p* = 0.659), summer (Pearson r = −0.121, *p* = 0.202), or autumn (Pearson r = 0.105, *p* = 0.288) (Figure 7a–c). Interestingly, 25(OH)D in winter, which was the lowest among all seasons, was not statically different but exhibited a subtle negative correlation with the Th1/Th2 cell ratio (Pearson r = −0.14, *p* = 0.14 (Figure 7d).

## 4. Discussion

Our study revealed that the vitamin D level is lower than sufficient (<30 ng/mL) in most reproductive-aged women with infertility (94%) visiting Kameda IVF Makuhari in Chiba (35.607_N), which is 45 km from Tokyo, Japan, and 57% were vitamin D-deficient (<20 ng/mL). A high prevalence of vitamin D insufficiency or deficiency has been reported in almost all age groups among males and females in Japan [30,40,41]. Furthermore, several studies have reported that the prevalence of vitamin D deficiency in Japanese women is high; however, these studies focused only on pregnant women [42,43,44,45,46,47,48,49,50]. To our knowledge, this is the first large cohort study to investigate vitamin D levels in reproductive-aged women with infertility in Japan, specifically investigating the association between 25(OH)D levels and ovarian function or implantation-related immunological markers.

Vitamin D deficiency or insufficiency is a major global health concern [51,52]. In Japan, the ROAD study, a large cohort study of 1088 women, reported that up to 81.3% of the cohort had serum 25(OH)D concentrations ≤ 30 ng/mL. Similarly, Tamaki et al. reported that only 10% of participants in 1211 women aged ≥50 years had blood 25OHD levels ≥ 30 ng/mL [53]. In contrast, only two reports are available on serum 25(OH)D concentrations in younger women who were not pregnant. One was a small study involving 77 Japanese female junior college students aged 19–24 years, which reported a mean serum 25(OH)D concentration of 13.6 ng/mL [54]. Another study reported that young Japanese women (*n* = 296, mean age: 21.2 ± 2.3 years) had a mean serum 25(OH)D concentration of 18.4 ± 4.9 ng/mL [55]. Chu et al. recently reported that a relatively large cohort of healthy reproductive-aged women (*n* = 351, median age: 28.0 years) in the Berlin Birth Cohort study had a mean serum 25(OH)D concentration of 18.37 ng/mL, and 57.3% of participants (*n* = 201) had 25(OH)D levels < 20 ng/mL [56]. In the present study, 2029 reproductive-aged women with infertility were reviewed; the participants had a mean serum 25(OH)D concentration of 18.2 ± 7.0 ng/mL, and rates of vitamin D deficiency/insufficiency were 57% and 28%, respectively. Although the findings of this study are consistent with those of previous reports in Japan and other countries [54,55,56,57], this study was the largest examining the association between vitamin D and reproductive-aged women with infertility, the number of whom has been increasing worldwide [58] and in Japan [59].

Serum 25(OH)D concentrations reflect not only vitamin D intake but also its production in skin, which is facilitated by ultraviolet (UV) irradiation [60]. As Japan has marked seasonal variations in climate and regional differences in latitude, a study covering multiple seasons and residential latitudes is needed. Previous studies from Japan have reported serum 25(OH)D concentrations in summer and winter with conflicting results, such as a significant correlation of serum 25(OH)D with UV exposure time and UV energy only in summer [61,62]. In this study, serum 25(OH)D levels in summer were the highest, and serum 25(OH)D levels in winter were the lowest. The seasonal pattern was similar to that of studies conducted in Kyoto, Japan, which is located at 35.011°N [50,63]. However, the results of some studies were not consistent with this study. In Tokushima, Japan, which is located at 35.040°N, serum 25(OH)D in autumn was the highest, and serum 25(OH)D in winter was the lowest [47]. Additionally, serum 25(OH)D in summer was the highest and serum 25(OH)D in spring was the lowest in Sapporo, Japan, which is located at 43.066°N [64]. The seasonal serum 25(OH)D level in Japan is at its lowest in winter. The median concentrations of the monthly measurements in Figure 4 show that the maximum values were reached in August. The minimum monthly values were recorded in February. Serum 25(OH)D levels were correlated with daylight, as previously reported [65]. However, there is a controversial association between serum 25(OH)D levels and daylight in December. There are regional food habits in Japan; indeed, there was a well-designed cohort study in Niigata Prefecture in which 25(OH)D levels were affected by a regional food habit of consuming salmon, which enriched the participants’ vitamin D intake from November to January [30]. In this study, we could not explain why seasonal 25(OH)D levels varied except daylight because there were no regional characteristics regarding dietary habits in Chiba which could influence serum 25(OH)D levels.

In recent years, several biological mechanisms have been postulated for the potential influence of vitamin D on pregnancy rates and live births [66,67]. Several studies on spontaneous pregnancy and serum vitamin D levels have recently provided conflicting results. Two studies reported that higher vitamin D levels were associated with modulating fertility and resulting in an increasing pregnancy rate [68,69], whereas two others suggested no marked association between serum vitamin D levels and t fertility and pregnancy outcomes [70,71]. Therefore, it may be hypothesized that vitamin D status is more closely associated with reproductive outcomes such as spontaneous pregnancy among reproductive-aged women with infertility, although further studies are required to confirm these findings. While early studies have demonstrated that vitamin D may be associated with ovarian function markers, such as AMH and basal FSH [72], the evidence has been conflicting. The most recent meta-analysis conducted by Moridi et al. in 2020 [73] showed that serum vitamin D levels were not associated with AMH levels, which may be due to the heterogeneity of the study population and the apparently complex relationship that may exist between vitamin D and AMH. In this study, we performed a large-scale analysis of the association between serum vitamin D and ovarian reserve markers, such as AMH and basal FSH, and found no associations. Vitamin D may influence endometrial receptivity by regulating focal immune reactions and play a pivotal role in embryo implantation [74]. In the focal endometrium, vitamin D may promote implantation via the inhibition of Th1 cell proliferation and by functionally and numerically promoting Th2 cells to regulate T-helper cell populations, resulting in a predominance of Th2 polarization [23,75]. In this study, we analyzed the associations between the Th1/Th2 ratio and seasonal serum 25(OH)D levels or the classification of serum 25(OH)D levels and found no associations. Taken together, vitamin D status in reproductive-aged women with infertility was not influenced by reproductive markers of ovarian function or implantation-related immunological markers.

Despite data calling into question the accuracy of vitamin D measurement and, consequently, the ability to determine vitamin D deficiency and potentially the susceptibility to poor ART outcomes, vitamin D measurement and supplementation is considered a relevant RIF intervention by published guidelines and is widely applied in clinical practice [76]. On the other hand, very recent guidelines and practice recommendations from the ESHRE [77] were published and commented following: (1) there are insufficient data to recommend the routine measurement of vitamin D levels or the treatment of vitamin D deficiency. (2) vitamin D measurement was baselessly considered a marker for RIF, although vitamin D supplementation used to be thoughtlessly used to intervene in RIF because the pressure on clinicians to intervene in cases involving RIF is considerable. However, we should reconsider whether the intervention with vitamin D is necessary or not. Additionally, an ESHRE-evidence-based guideline on unexplained infertility mentioned that no role of vitamin D has been found, and the evidence of measurement and treatment with vitamin D is of relatively low quality but generally against specific testing outside of other medical or environmental indication [78]. In addition, they commented that vitamin D measurement and treatment is relatively inexpensive and widely available with simple dietary remediation [78]. However, this comment is an obscure and ambiguous because that comments is not completely denied the issue of vitamin D, and may cause clinicians and patients to misunderstand whether vitamin D measurement and supplementation are recommended or not. Hence, we agree with those guidelines and recommendations but still believe that clinical vitamin D measurement and supplementation should be continued in order to avoid patient confusion. However, we need to take the opportunity to re-evaluate whether this is really necessary in the future.

This study had some limitations. First, we did not investigate vitamin D intake using detailed questionnaires, such as the “Food Frequency Questionnaire (FFQ)” [79]. Second, the non-randomized cross-sectional nature of this study limits the generalizability of the results. The lack of standardized lifestyle conditions, such as clothing style, regular use of sunscreen, sunlight exposure, and socioeconomic status, were considered potential confounders. Third, reproductive-aged women without infertility as controls should essentially be set up and analyzed to compare. However, this study was a cross-sectional study of only reproductive-aged women with infertility, and the absence of a control group is a limitation. Fourth, this study demonstrated the absence of seasonal variability by analyzing AMH and FSH as indicators of ovarian function and Th1/Th2 as immunological implantation markers. However, a wide variety of ovarian functional markers [80] and immunological implantation markers [81] have been reported, and the limitation is that all markers were not analyzed. The strengths of this study are its large sample size and the stringent inclusion criteria used to eliminate confounders, such as BMI and smoking, that might have affected serum 25(OH)D concentration. Furthermore, we evaluated not only serum AMH levels in relation to 25(OH)D but also other important markers such as basal FSH levels and the Th1/Th2 ratio.

## 5. Conclusions

We determined the serum 25(OH)D concentration in reproductive-aged women with infertility in Japan and investigated the association between serum 25(OH)D concentration and ovarian function and implantation-related immunological markers. Vitamin D insufficiency was common in our cohort and showed a strong seasonal effect, with the lowest values observed in winter, especially in February. In addition, adequate vitamin D levels are achieved in only 6% of reproductive-aged women with infertility in Japan. These seasonal variations should be considered when measuring serum vitamin D levels and may also have implications for current medical and dietary recommendations. Vitamin D supplementation may be necessary for pre-conception care throughout the year, especially in winter. As the results of future research on vitamin D and reproduction accumulate with well-designed interventional studies, a re-examination of the current periconceptual screening and treatment guidelines may be warranted.

## Figures and Tables

**Table 1:** Characteristics of women with infertility (*n* = 2029).

	Means ± SDs or Percentage	Range
Age (years)	34.5 ± 4.5	22–47
Gravity	0.5 ± 0.9	0–8
Parity	0.2 ± 0.5	0–4
BMI (kg/m^2^)	21.6 ± 3.5	14.8–40.9
Duration of infertility (months)	22.7 ± 18.5	0–120
Causal factors of infertility, % (*n*)		
Reduced ovarian reserve	20.7 (421)	
Ovulation disorder	24.1 (488)	
Uterine factor	40.8 (827)	
Tubal factor	18.2 (369)	
Endometriosis	11.7 (238)	
Male factor	42.2 (857)	
Unexplained	14.6 (297)	
Day 3 serum FSH (IU/mL)	8.2 ± 5.2	0.1–85.9
Day 3 serum LH	7.4 ± 5.7	0.1–61.4
Day 3 serum estrogen (E2)	50.8 ± 59.8	5.0–354
AMH (ng/mL)	3.6 ± 3.1	0.2–48.2
Thyroid-stimulating hormone (TSH) (μIU/mL)	1.7 ± 1.1	0.1–13.5
Free Thyroxine (FT4) (ng/dL)	1.3 ± 0.2	0.5–3.8
Vitamin D serum levels (ng/mL)	18.2 ± 7.0	4.1–46.6
Vitamin D supplement user, % (*n*)	10.8 (220)	
Current smoker, % (*n*)	15.2 (320)	
Occupation, % (*n*)		
Employee	66.3 (1346)	
Part-time	11.5 (234)	
Housewife	14.5 (295)	
Out of work	2.9 (58)	

**Table 2:** Factors associated with 25(OH) vitamin D level.

		*n*	Means ± SD (ng/mL)	Median (IQR) (ng/mL)	*p*-Value	
					Kruskal–Wallis Test	Jonckheere–Terpstra Test
Age, years	<25	11	15.5 ± 5.2	15.2 (11.2–18.5)	0.235	
	25–29	229	18.4 ± 7.6	16.2 (13.1–20.1)		
	30–34	651	17.9 ± 6.8	16.9 (12.9–21.9)		
	35–39	576	18.3 ± 6.9	17.5 (13.3–22.2)		
	≥40	280	18.1 ± 7.3	16.5 (12.4–22.5)		
BMI (kg/m^2^)	<18.35	285	18.4 ± 7.6	17.0 (13.2–22.3)	0.88	0.675
	≥18.5–<25	1376	18.2 ± 7.0	17.1 (13.1–22.4)		
	≥25	279	17.8 ± 6.2	16.8 (13.1–21.5)		
AMH (ng/mL)	Q1 (<1.57)	435	18.4 ± 7.0	17.5 (13.2–22.5)	0.336	0.195
	Q2 (1.57–2.985)	438	17.7 ± 6.8	16.6 (12.9–21.7)		
	Q3 (2.986–4.9374)	436	18.1 ± 7.1	16.8 (13.1–21.5)		
	Q4 (≥4.9375)	437	17.5 ± 6.5	16.8 (12.9–21.2)		
FSH (IU/mL)	Q1 (<6.3)	480	18.0 ± 6.9	1.7 (12.8–21.6)	0.241	0.0902
	Q2 (6.3–7.3)	477	17.8 ± 6.9	16.6 (12.7–21.9)		
	Q3 (7.4–8.7)	476	18.5 ± 7.0	17.4 (13.5–22.2)		
	Q4 (≥8.8)	490	18.5 ± 7.1	17.4 (13.1–23.0)		
TSH (μIU/mL)	<2.5	1382	18.1 ± 6.9	17.1 (0.94–1.72)	0.981	0.8697
	2.5–4.9	278	18.0 ± 6.8	16.8 (2.74–4.88)		
	≥5.0	33	18.6 ± 9.0	16.4 (5.34–7.19)		
Season of blood collection	Spring	504	18.2 ± 7.7	16.5 (12.6–22.4)	0.00000000439	
	Summer	490	19.0 ± 6.4	18.2 (14.1–22.7)		
	Autumn	561	18.7 ± 6.6	18.1 (13.9–22.6)		
	Winter	474	16.9 ± 7.2	15.3 (11.5–20.6)		

IQR: interquartile range.

## Data Availability

The datasets generated and/or analyzed during this current study are not publicly available due to participant privacy, but they are available from the corresponding author upon reasonable request.

## References

[1] Holick M.F. (2007). Vitamin D deficiency. N. Engl. J. Med..

[2] Souberbielle J.-C., Body J.-J., Lappe J.M., Plebani M., Shoenfeld Y., Wang T.J., Bischoff-Ferrari H.A., Cavalier E., Ebeling P.R., Fardellone P. (2010). Vitamin D and musculoskeletal health, cardiovascular disease, autoimmunity and cancer: Recommendations for clinical practice. Autoimmun. Rev..

[3] Luk J., Torrealday S., Neal Perry G., Pal L. (2012). Relevance of vitamin D in reproduction. Hum. Reprod..

[4] Jukic A.M., Steiner A.Z., Baird D.D. (2015). Association between serum 25-hydroxyvitamin D and ovarian reserve in premenopausal women. Menopause.

[5] Dennis N.A., Houghton L.A., Jones G.T., van Rij A.M., Morgan K., McLennan I.S. (2012). The level of serum anti-Müllerian hormone correlates with vitamin D status in men and women but not in boys. J. Clin. Endocrinol. Metab..

[6] Dabrowski F.A., Grzechocinska B., Wielgos M. (2015). The Role of Vitamin D in Reproductive Health—A Trojan Horse or the Golden Fleece?. Nutrients.

[7] Butts S.F., Seifer D.B., Koelper N., Senapati S., Sammel M.D., Hoofnagle A.N., Kelly A., Krawetz S.A., Santoro N., Zhang H. (2019). Vitamin D Deficiency Is Associated With Poor Ovarian Stimulation Outcome in PCOS but Not Unexplained Infertility. J. Clin. Endocrinol. Metab..

[8] Zhao J., Liu S., Wang Y., Wang P., Qu D., Liu M., Ma W., Li Y. (2019). Vitamin D improves in-vitro fertilization outcomes in infertile women with polycystic ovary syndrome and insulin resistance. Minerva Med..

[9] Ota K., Dambaeva S., Han A.-R., Beaman K., Gilman-Sachs A., Kwak-Kim J. (2014). Vitamin D deficiency may be a risk factor for recurrent pregnancy losses by increasing cellular immunity and autoimmunity. Hum. Reprod..

[10] Ota K., Dambaeva S., Kim M.W.I., Han A.R., Fukui A., Gilman-Sachs A., Beaman K., Kwak-Kim J. (2015). 1,25-Dihydroxy-vitamin D3 regulates NK-cell cytotoxicity, cytokine secretion, and degranulation in women with recurrent pregnancy losses. Eur. J. Immunol..

[11] Rajaei S., Mirahmadian M., Jeddi-Tehrani M., Tavakoli M., Zonoobi M., Dabbagh A., Zarnani A.H. (2012). Effect of 1,25(OH)2 vitamin D3 on cytokine production by endometrial cells of women with repeated implantation failure. Gynecol. Endocrinol..

[12] Lemire J.M. (1995). Immunomodulatory actions of 1,25-dihydroxyvitamin D3. J. Steroid Biochem. Mol. Biol..

[13] D’Ambrosio D., Cippitelli M., Cocciolo M.G., Mazzeo D., Di Lucia P., Lang R., Sinigaglia F., Panina-Bordignon P. (1998). Inhibition of IL-12 production by 1,25-dihydroxyvitamin D3. Involvement of NF-kappaB downregulation in transcriptional repression of the p40 gene. J. Clin. Investig..

[14] Cantorna M.T., Hayes C.E., DeLuca H.F. (1996). 1,25-Dihydroxyvitamin D3 reversibly blocks the progression of relapsing encephalomyelitis, a model of multiple sclerosis. Proc. Natl. Acad. Sci. USA.

[15] Cantorna M.T., Humpal-Winter J., DeLuca H.F. (2000). In vivo upregulation of interleukin-4 is one mechanism underlying the immunoregulatory effects of 1,25-dihydroxyvitamin D3. Arch. Biochem. Biophys..

[16] Das M., Tomar N., Sreenivas V., Gupta N., Goswami R. (2014). Effect of vitamin D supplementation on cathelicidin, IFN-γ, IL-4 and Th1/Th2 transcription factors in young healthy females. Eur. J. Clin. Nutr..

[17] Kwak-Kim J., Skariah A., Wu L., Salazar D., Sung N., Ota K. (2016). Humoral and cellular autoimmunity in women with recurrent pregnancy losses and repeated implantation failures: A possible role of vitamin D. Autoimmun. Rev..

[18] Raghupathy R., Makhseed M., Azizieh F., Omu A., Gupta M., Farhat R. (2000). Cytokine production by maternal lymphocytes during normal human pregnancy and in unexplained recurrent spontaneous abortion. Hum. Reprod..

[19] Ng S.C., Gilman-Sachs A., Thaker P., Beaman K.D., Beer A.E., Kwak-Kim J. (2002). Expression of intracellular Th1 and Th2 cytokines in women with recurrent spontaneous abortion, implantation failures after IVF/ET or normal pregnancy. Am. J. Reprod. Immunol..

[20] Mekinian A., Cohen J., Alijotas-Reig J., Carbillon L., Nicaise-Roland P., Kayem G., Daraï E., Fain O., Bornes M. (2016). Unexplained Recurrent Miscarriage and Recurrent Implantation Failure: Is There a Place for Immunomodulation?. Am. J. Reprod. Immunol..

[21] Kwak-Kim J.Y.H., Chung-Bang H.S., Ng S.C., Ntrivalas E.I., Mangubat C.P., Beaman K.D., Beer A.E., Gilman-Sachs A. (2003). Increased T helper 1 cytokine responses by circulating T cells are present in women with recurrent pregnancy losses and in infertile women with multiple implantation failures after IVF. Hum. Reprod..

[22] Lim K.J., Odukoya O.A., Ajjan R.A., Li T.C., Weetman A.P., Cooke I.D. (2000). The role of T-helper cytokines in human reproduction. Fertil. Steril..

[23] Ikemoto Y., Kuroda K., Nakagawa K., Ochiai A., Ozaki R., Murakami K., Jinushi M., Matsumoto A., Sugiyama R., Takeda S. (2018). Vitamin D Regulates Maternal T-Helper Cytokine Production in Infertile Women. Nutrients.

[24] Bouillon R., Carmeliet G. (2018). Vitamin D insufficiency: Definition, diagnosis and management. Best. Pract. Res. Clin. Endocrinol. Metab..

[25] Holick M.F., Binkley N.C., Bischoff-Ferrari H.A., Gordon C.M., Hanley D.A., Heaney R.P., Murad M.H., Weaver C.M. (2012). Guidelines for preventing and treating vitamin D deficiency and insufficiency revisited. J. Clin. Endocrinol. Metab..

[26] Liu X., Baylin A., Levy P.D. (2018). Vitamin D deficiency and insufficiency among US adults: Prevalence, predictors and clinical implications. Br. J. Nutr..

[27] Herrick K.A., Storandt R.J., Afful J., Pfeiffer C.M., Schleicher R.L., Gahche J.J., Potischman N. (2019). Vitamin D status in the United States, 2011–2014. Am. J. Clin. Nutr..

[28] Liu W., Hu J., Fang Y., Wang P., Lu Y., Shen N. (2021). Vitamin D status in Mainland of China: A systematic review and meta-analysis. EClinicalMedicine.

[29] Hu Y., Chen J., Wang R., Li M., Yun C., Li W., Yang Y., Piao J., Yang X., Yang L. (2017). Vitamin D nutritional status and its related factors for Chinese children and adolescents in 2010–2012. Nutrients.

[30] Nakamura K., Kitamura K., Takachi R., Saito T., Kobayashi R., Oshiki R., Watanabe Y., Tsugane S., Sasaki A., Yamazaki O. (2015). Impact of demographic, environmental, and lifestyle factors on vitamin D sufficiency in 9084 Japanese adults. Bone.

[31] Yoshimura N., Muraki S., Oka H., Morita M., Yamada H., Tanaka S., Kawaguchi H., Nakamura K., Akune T. (2013). Profiles of vitamin D insufficiency and deficiency in Japanese men and women: Association with biological, environmental, and nutritional factors and coexisting disorders: The ROAD study. Osteoporos. Int..

[32] Laganà A.S., Vitale S.G., Ban Frangež H., Vrtačnik-Bokal E., D’Anna R. (2017). Vitamin D in human reproduction: The more, the better? An evidence-based critical appraisal. Eur. Rev. Med. Pharmacol. Sci..

[33] Evers J.L.H. (2002). Female subfertility. Lancet.

[34] Okazaki R., Ozono K., Fukumoto S., Inoue D., Yamauchi M., Minagawa M., Michigami T., Takeuchi Y., Matsumoto T., Sugimoto T. (2017). Assessment criteria for vitamin D deficiency/insufficiency in Japan: Proposal by an expert panel supported by the Research Program of Intractable Diseases, Ministry of Health, Labour and Welfare, Japan, the Japanese Society for Bone and Mineral Research and the Japan Endocrine Society [Opinion]. J. Bone Min. Metab..

[35] Nakagawa K., Kwak-Kim J., Ota K., Kuroda K., Hisano M., Sugiyama R., Yamaguchi K. (2015). Immunosuppression with tacrolimus improved reproductive outcome of women with repeated implantation failure and elevated peripheral blood TH1/TH2 cell ratios. Am. J. Reprod. Immunol..

[36] Steinman L. (2007). A brief history of TH17, the first major revision in the TH1/TH2 hypothesis of T cell–mediated tissue damage. Nat. Med..

[37] Kanda Y. (2013). Investigation of the freely available easy-to-use software ‘EZR’ for medical statistics. Bone Marrow Transpl..

[38] Holick M.F., Binkley N.C., Bischoff-Ferrari H.A., Gordon C.M., Hanley D.A., Heaney R.P., Murad M.H., Weaver C.M. (2011). Evaluation, treatment, and prevention of vitamin D deficiency: An Endocrine Society clinical practice guideline. J. Clin. Endocrinol. Metab..

[39] Del Valle H.B., Yaktine A.L., Taylor C.L., Ross A.C. (2011). Dietary Reference Intakes for Calcium and Vitamin D.

[40] Akter S., Eguchi M., Kurotani K., Kochi T., Kashino I., Ito R., Kuwahara K., Tsuruoka H., Kabe I., Mizoue T. (2017). Serum 25-hydroxyvitamin D and metabolic syndrome in a Japanese working population: The Furukawa Nutrition and Health Study. Nutrition.

[41] Tsugawa N., Uenishi K., Ishida H., Ozaki R., Takase T., Minekami T., Uchino Y., Kamao M., Okano T. (2016). Association between vitamin D status and serum parathyroid hormone concentration and calcaneal stiffness in Japanese adolescents: Sex differences in susceptibility to vitamin D deficiency. J. Bone Miner. Metab..

[42] Shibata M., Suzuki A., Sekiya T., Sekiguchi S., Asano S., Udagawa Y., Itoh M. (2011). High prevalence of hypovitaminosis D in pregnant Japanese women with threatened premature delivery. J. Bone Miner. Metab..

[43] Shiraishi M., Haruna M., Matsuzaki M., Murayama R. (2014). Demographic and lifestyle factors associated with vitamin D status in pregnant Japanese women. J. Nutr. Sci. Vitaminol..

[44] Shiraishi M., Haruna M., Matsuzaki M., Murayama R., Kitanaka S., Sasaki S. (2015). Validity of a self-administered diet history questionnaire for estimating vitamin D intakes of J apanese pregnant women. Matern. Child Nutr..

[45] Yonetani N., Kaji T., Hichijo A., Nakayama S., Maeda K., Irahara M. (2018). Effect of prolonged hospitalization for threatened preterm labor on maternal and fetal vitamin D levels. J. Obstet. Gynaecol. Res..

[46] Kanatani K.T., Nakayama T., Adachi Y., Hamazaki K., Onishi K., Konishi Y., Kawanishi Y., Go T., Sato K., Kurozawa Y. (2019). High frequency of vitamin D deficiency in current pregnant Japanese women associated with UV avoidance and hypo-vitamin D diet. PLoS ONE.

[47] Sogawa E., Kaji T., Nakayama S., Yoshida A., Yonetani N., Maeda K., Yasui T., Irahara M. (2019). Seasonal variation of serum 25 (OH) vitamin D levels in maternal and umbilical cord blood in Japanese women. J. Med. Investig..

[48] Yoshikata H., Tsugawa N., Watanabe Y., Tsuburai T., Chaki O., Hirahara F., Miyagi E., Sakakibara H., Uenishi K., Okano T. (2020). 25-Hydroxyvitamin D profiles and maternal bone mass during pregnancy and lactation in Japanese women. J. Bone Miner. Metab..

[49] Takaoka N., Nishida K., Sairenchi T., Umesawa M., Noguchi R., Someya K., Kobashi G. (2020). Changes in vitamin D status considering hemodilution factors in Japanese pregnant women according to trimester: A longitudinal survey. PLoS ONE.

[50] Yamade I., Inoue T., Hamada H., Sudou S., Otsubo M., Sawada M., Nakayama T., Hatayama H. (2021). Ineffectiveness of antenatal guidance intervention for vitamin D insufficiency and deficiency in pregnant women in Kyoto, Japan. J. Obs. Gynaecol. Res..

[51] Mithal A., Wahl D.A., Bonjour J.P., Burckhardt P., Dawson-Hughes B., Eisman J.A., El-Hajj Fuleihan G., Josse R.G., Lips P., Morales-Torres J. (2009). Global vitamin D status and determinants of hypovitaminosis D. Osteoporos. Int..

[52] Hilger J., Friedel A., Herr R., Rausch T., Roos F., Wahl D.A., Pierroz D.D., Weber P., Hoffmann K. (2014). A systematic review of vitamin D status in populations worldwide. Br. J. Nutr..

[53] Tamaki J., Iki M., Sato Y., Kajita E., Nishino H., Akiba T., Matsumoto T., Kagamimori S. (2017). Total 25-hydroxyvitamin D levels predict fracture risk: Results from the 15-year follow-up of the Japanese Population-based Osteoporosis (JPOS) Cohort Study. Osteoporos. Int..

[54] Nakamura K., Nashimoto M., Tsuchiya Y., Obata A., Miyanishi K., Yamamoto M. (2001). Vitamin D insufficiency in Japanese female college students: A preliminary report. Int. J. Vitam. Nutr. Res..

[55] Ohta H., Kuroda T., Tsugawa N., Onoe Y., Okano T., Shiraki M. (2018). Optimal vitamin D intake for preventing serum 25-hydroxyvitamin D insufficiency in young Japanese women. J. Bone Min. Metab..

[56] Chu C., Tsuprykov O., Chen X., Elitok S., Krämer B.K., Hocher B. (2021). Relationship Between Vitamin D and Hormones Important for Human Fertility in Reproductive-Aged Women. Front. Endocrinol..

[57] Horton-French K., Dunlop E., Lucas R.M., Pereira G., Black L.J. (2021). Prevalence and predictors of vitamin D deficiency in a nationally representative sample of Australian adolescents and young adults. Eur. J. Clin. Nutr..

[58] Dyer S., Chambers G.M., de Mouzon J., Nygren K.G., Zegers-Hochschild F., Mansour R., Ishihara O., Banker M., Adamson G.D. (2016). International Committee for Monitoring Assisted Reproductive Technologies world report: Assisted Reproductive Technology 2008, 2009 and 2010. Hum. Reprod..

[59] Ishihara O., Jwa S.C., Kuwahara A., Katagiri Y., Kuwabara Y., Hamatani T., Harada M., Osuga Y. (2021). Assisted reproductive technology in Japan: A summary report for 2018 by the Ethics Committee of the Japan Society of Obstetrics and Gynecology. Reprod. Med. Biol..

[60] Hossein-nezhad A., Holick M.F. (2013). Vitamin D for health: A global perspective. Mayo. Clin. Proc..

[61] Asakura K., Etoh N., Imamura H., Michikawa T., Nakamura T., Takeda Y., Mori S., Nishiwaki Y. (2020). Vitamin D Status in Japanese Adults: Relationship of Serum 25-Hydroxyvitamin D with Simultaneously Measured Dietary Vitamin D Intake and Ultraviolet Ray Exposure. Nutrients.

[62] Kuwabara A., Tsugawa N., Mizuno K., Ogasawara H., Watanabe Y., Tanaka K. (2019). A simple questionnaire for the prediction of vitamin D deficiency in Japanese adults (Vitaimn D Deficiency questionnaire for Japanese: VDDQ-J). J. Bone Min. Metab..

[63] Ono Y., Suzuki A., Kotake M., Zhang X., Nishiwaki-Yasuda K., Ishiwata Y., Imamura S., Nagata M., Takamoto S., Itoh M. (2005). Seasonal changes of serum 25-hydroxyvitamin D and intact parathyroid hormone levels in a normal Japanese population. J. Bone Miner. Metab..

[64] Niino M., Fukazawa T., Miyazaki Y., Ura S., Takahashi E., Minami N., Akimoto S., Amino I., Naganuma R., Kikuchi S. (2021). Seasonal fluctuations in serum levels of vitamin D in Japanese patients with multiple sclerosis. J. Neuroimmunol..

[65] Klenk J., Rapp K., Denkinger M.D., Nagel G., Nikolaus T., Peter R., Koenig W., Böhm B.O., Rothenbacher D. (2013). Seasonality of vitamin D status in older people in Southern Germany: Implications for assessment. Age Ageing.

[66] Heyden E.L., Wimalawansa S.J. (2018). Vitamin D: Effects on human reproduction, pregnancy, and fetal well-being. J. Steroid Biochem. Mol. Biol..

[67] Fichera M., Török P., Tesarik J., Della Corte L., Rizzo G., Garzon S., Carlea A., Di Angelo Antonio S., Zito G., Panella M.M. (2020). Vitamin D, reproductive disorders and assisted reproduction: Evidences and perspectives. Int. J. Food Sci. Nutr..

[68] Mumford S.L., Garbose R.A., Kim K., Kissell K., Kuhr D.L., Omosigho U.R., Perkins N.J., Galai N., Silver R.M., Sjaarda L.A. (2018). Association of preconception serum 25-hydroxyvitamin D concentrations with livebirth and pregnancy loss: A prospective cohort study. Lancet Diabetes Endocrinol..

[69] Fung J.L., Hartman T.J., Schleicher R.L., Goldman M.B. (2017). Association of vitamin D intake and serum levels with fertility: Results from the Lifestyle and Fertility Study. Fertil. Steril..

[70] Somigliana E., Paffoni A., Lattuada D., Colciaghi B., Filippi F., La Vecchia I., Tirelli A., Baffero G.M., Persico N., Viganò P. (2016). Serum levels of 25-hydroxyvitamin D and time to natural pregnancy. Gynecol. Obstet. Investig..

[71] Møller U., Streym S., Heickendorff L., Mosekilde L., Rejnmark L. (2012). Effects of 25OHD concentrations on chances of pregnancy and pregnancy outcomes: A cohort study in healthy Danish women. Eur. J. Clin. Nutr..

[72] Irani M., Merhi Z. (2014). Role of vitamin D in ovarian physiology and its implication in reproduction: A systematic review. Fertil. Steril..

[73] Moridi I., Chen A., Tal O., Tal R. (2020). The Association between Vitamin D and Anti-Müllerian Hormone: A Systematic Review and Meta-Analysis. Nutrients.

[74] Evans K.N., Nguyen L., Chan J., Innes B.A., Bulmer J.N., Kilby M.D., Hewison M. (2006). Effects of 25-Hydroxyvitamin D3 and 1,25-Dihydroxyvitamin D3 on Cytokine Production by Human Decidual Cells1. Biol. Reprod..

[75] Boonstra A., Barrat F.J., Crain C., Heath V.L., Savelkoul H.F., O’Garra A. (2001). 1α,25-Dihydroxyvitamin D3 has a direct effect on naive CD4+ T cells to enhance the development of Th2 cells. J. Immunol..

[76] Cimadomo D., Craciunas L., Vermeulen N., Vomstein K., Toth B. (2021). Definition, diagnostic and therapeutic options in recurrent implantation failure: An international survey of clinicians and embryologists. Hum. Reprod..

[77] Cimadomo D., de los Santos M.J., Griesinger G., Lainas G., Le Clef N., McLernon D.J., Montjean D., Toth B., Vermeulen N., ESHRE Working Group on Recurrent Implantation Failure (2023). ESHRE good practice recommendations on recurrent implantation failure. Hum. Reprod. Open.

[78] Romualdi D., Ata B., Bhattacharya S., Bosch E., Costello M., Gersak K., Homburg R., Mincheva M., Norman R.J., he Guideline Group on Unexplained Infertility (2023). Evidence-based guideline: Unexplained infertility. Hum. Reprod..

[79] Ishihara J., Inoue M., Kobayashi M., Tanaka S., Yamamoto S., Iso H., Tsugane S. (2006). Impact of the revision of a nutrient database on the validity of a self-administered food frequency questionnaire (FFQ). J. Epidemiol..

[80] Penzias A., Azziz R., Bendikson K., Falcone T., Hansen K., Hill M., Hurd W., Jindal S., Kalra S., Mersereau J. (2020). Testing and interpreting measures of ovarian reserve: A committee opinion. Fertil. Steril..

[81] Mrozikiewicz A.E., Ożarowski M., Jędrzejczak P. (2021). Biomolecular Markers of Recurrent Implantation Failure—A Review. Int. J. Mol. Sci..

